# Myocarditis in the Setting of Recent COVID-19 Vaccination

**DOI:** 10.1155/2021/6806500

**Published:** 2021-10-19

**Authors:** Laura Onderko, Benjamin Starobin, Amy E. Riviere, Patrick K. Hohl, Colin T. Phillips, Roisin B. Morgan, Aimee Welsh, Sanjeev A. Francis, Maxwell Eyram Afari

**Affiliations:** Cardiovascular Disease Service Line, Maine Medical Center, Portland, Maine, USA

## Abstract

We report three patients who presented with chest pain after receiving either the BNT162b2 Pfizer/BioNTech or mRNA-1273 Moderna/NIH vaccine. Clinical presentation, biomarker, and cardiac MRI supported myocarditis. It is imperative that potential side effects of COVID-19 vaccine are reported to improve our knowledge about COVID-19 and mRNA vaccines.

## 1. Introduction

The severe acute respiratory syndrome coronavirus 2 (SARS-CoV-2) is the causative agent of the Coronavirus Disease-2019 (COVID-19) pandemic. The global impact of the COVID-19 pandemic continues since initial descriptions of the COVID-19 syndrome in December 2019. Rapid development and administration of COVID-19 vaccines promise to mitigate COVID-19 fatalities. Early studies show that vaccination against COVID-19 is highly effective and safe [1]. We present three cases of myocarditis that occurred with close temporal relationship to recent COVID-19 vaccine administration with three cases of male patients aged 25-36 years diagnosed with myocarditis several days after receiving the second mRNA vaccine.

## 2. Case Reports

### 2.1. Case 1

A 25-year-old male with well-controlled Crohn's disease (not prescribed any medications) presented to the emergency department with chest pain. The chest pain began the day prior to presentation, worse with inspiration and with associated jaw pain. He reported that he received his second dose of the COVID-19 vaccine (BNT162b2 Pfizer/BioNTech) four days prior to presentation. He experienced malaise and myalgias that resolved within twenty-four hours after receiving the vaccine. Labs were significant for a Troponin T that peaked at 0.74 ng/ml (reference < 0.2 ng/ml). Tick panel (for Lyme, anaplasmosis, babesiosis, and ehrlichiosis) was negative since these are part of the differential work-up of myocarditis in the New England region of the United States. His electrocardiogram did not show any ischemic changes, and echocardiogram was significant for low normal left ventricular ejection fraction (LVEF) of 50%. He was admitted for further work-up and started on colchicine and ibuprofen for presumed myopericarditis. He underwent cardiac MRI which showed a left ventricular fraction of 56% and findings consistent with myocarditis with myocardial edema seen by T2 mapping and myocardial injury with focal late gadolinium enhancement (LGE) with multiple foci in the midwall. He did not have evidence of pericardial inflammation. He did not undergo endomyocardial biopsy. COVID-19 polymerase chain reaction (PCR) was negative. During his hospital stay, he was monitored on telemetry without any arrhythmias observed. He was discharged on low-dose beta-blocker with plans for repeat outpatient echocardiogram and cardiac monitor to screen for any arrhythmias as well as activity restriction.

### 2.2. Case 2

**Figure 1:**
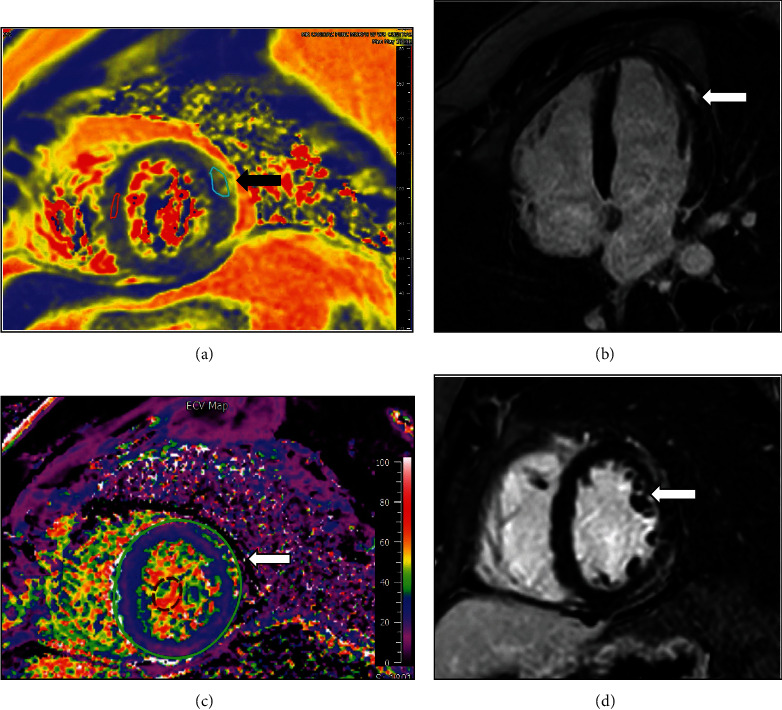
Cardiac MRI of patient C: (a) T2 map demonstrating increased T2 signal consistent with edema in epicardium of the apical lateral wall; (b) four-chamber view showing late gadolinium enhancement in the apical lateral wall; (c) ECV map showing increased signal consistent with edema in the midanterolateral segments; (d) short axis with late gadolinium enhancements in the midanterolateral segments.

A 28-year-old male with a medical history significant for obesity presented with chest pain and lateral ST elevation myocardial infarction by electrocardiogram. He was taken to the catheterization laboratory, and coronary angiogram showed no luminal irregularities. On interview, he reported receiving his second dose of COVID-19 vaccine (NT162b2 Pfizer/BioNTech) three days prior to presentation. After receiving the vaccine, he experienced fatigue, chills, and myalgias that resolved the day before presentation. Labs were significant for Troponin T peak of 1.42 ng/ml, C-reactive protein (CRP) peak of 68.6 mg/l (reference < 8.0 mg/l), and Erythrocyte Sedimentation Rate (ESR) of 19 mm/h (reference < 15 mm/h). He underwent cardiac MRI which showed a left ventricular ejection fraction of 56% and evidence of myocarditis with myocardial edema demonstrated on T2 mapping and myocardial injury with focal LGE in the basal inferior, mid- to distal lateral/inferolateral walls (Figure 1). He also did not undergo endomyocardial biopsy, and COVID-19 PCR was negative. During his hospital stay, he was monitored on telemetry without any arrhythmias observed. He was discharged on low-dose beta blocker and activity restriction with plan for continued outpatient follow-up.

### 2.3. Case 3

**Figure 2:**
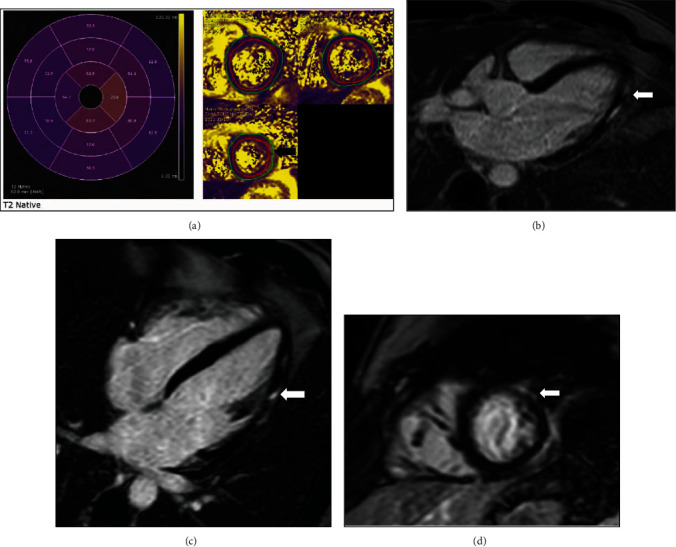
Patient D cardiac MRI: (a) T2 map demonstrating increased T2 signal consistent with edema in the epicardium of the apical lateral wall; (b–d) 3-chamber, 4-chamber, and apical short axis delayed images, respectively, demonstrating epicardial late gadolinium enhancement (LGE) corresponding to area of edema on T2 map.

A 36-year-old male with a past medical history significant for gastroesophageal reflux disease (not prescribed any medications) who presented with chest pain and lateral ST elevations consistent with ST elevation myocardial infarction. He was taken to the cardiac catheterization laboratory, and coronary angiogram showed no luminal irregularities. On interview, he reported receiving the second dose of the mRNA-1273 Moderna/NIH vaccine two days prior to presentation. His Troponin T peaked at 0.35 ng/ml, CRP 49.7 mg/l, and ESR of 24 mm/h. Echocardiogram showed a left ventricular ejection fraction of 55-60%. Cardiac MRI was again consistent with myocarditis with LVEF of 56% and myocardial edema by T2 mapping and myocardial injury with focal LGE in the mid- to distal inferolateral and lateral walls (Figure 2). Again, no endomyocardial biopsy was obtained, and COVID-19 PCR was negative. During his hospital stay, he was monitored on telemetry without any arrhythmias observed. He was also discharged with low-dose beta-blocker, advice on activity restrictions, and outpatient follow-up.

## 3. Discussion

We describe three cases of myocarditis in young, healthy patients following COVID-19 vaccine administration (Table 1). The temporal timing of our cases correlates with similar reports in literature of myocarditis occurring 12-96 hours following immunization with an mRNA COVID-19 vaccine [2, 3]. These cases demonstrate a similar temporal relationship, increasing the likelihood of causality. This safety signal was not clinically apparent in the early vaccine experience, raising the possibility that the association is purely coincidental as viruses responsible for myocarditis follow a seasonal distribution [4].

All three patients were ruled out for COVID-19 infection using the Centers for Disease Control and Prevention- (CDC-) approved gold standard test: polymerase chain reaction (PCR). Previous reports had demonstrated an association between COVID-19 infection and myocarditis [5]. Routine antigen or antibody testing is not part of our clinical work flow, and some have questioned the utility of antibody testing in the postvaccination cohort [6].

In case 1, we considered the possibility of Crohn's disease being a confounding factor for myocardial injury. On review of available case reports in the literature, episodes of myocarditis associated to Crohn's disease occur most often in the context of a disease flare or secondary to disease-modifying medication and remain an extremely rare extraintestinal manifestation of Crohn's disease [7].

Importantly, none of these patients developed malignant manifestations of myocarditis. They maintained normal systolic function and did not have any symptoms of clinical heart failure, arrhythmias, or conduction abnormalities. In addition, for the number of vaccinations that have been administered in our hospital catchment area, these cases would be exceedingly rare. At the time these patients presented to our institution, the Maine CDC reported vaccine administration of 661,051 initial doses and 612,781 second doses. Taking both these factors into consideration and knowing the morbidity and mortality of COVID-19 infection, the overall benefit of the COVID-19 vaccine outweighs this potential risk.

Proposed mechanisms of a COVID-19 vaccine-associated myocarditis are speculative. Both the BNT162b2 Pfizer/BioNTech and mRNA-1273 Moderna/NIH vaccines produced high-affinity neutralizing antibody in the early phases [8]. This raises the possibility of an inappropriate adaptive immune response causing clinical myocarditis. Serologic testing of the early phase I subjects demonstrated a robust T cell response, and specific cytokines were tracked [8]. It is plausible that the T cell immunity in these cases became dysregulated resulting in abnormal cytokine release. Another possible mechanism includes impaired development of T cell memory and subsequent exaggerated upregulation of cytokines [9]. Other considerations include a cross-reactivity of existing memory T cells from prior infections with other coronaviruses, which can result in a heterologous T cell memory with resultant exaggerated cytokine release [9].

The smallpox vaccine is linked to myocarditis. In 2002, recommendations were made for smallpox (variola virus) vaccination of enlisted military personnel as well as civilian health care workers. Of 730,580 armed forces personnel vaccinated with live vaccinia vaccine (Dryvax), 86 cases of myopericarditis occurred, at a rate 7.5 times higher than the expected background rate. In a subsequent prospective study of the ACAM2000 vaccine approved in 2007, 7 out of 1307 vaccinated individuals developed myocarditis with no fatalities. However, these vaccines do represent an entirely different model of vaccination as compared to COVID-19 vaccines given their use of live virus to generate an immune response [10].

## 4. Conclusion

There have been early reports of a presumed link between COVID-19 vaccination and subsequent development of myocarditis, but no causative link has been established. Our cases add to others where a temporal relationship between vaccination and myocarditis has been described. Ultimately, further exploration of this clinical phenomenon is warranted.

## Figures and Tables

**Table 1:** Patient characteristics and results.

Patient	Age	Sex	Medical history	Symptom onset	Vaccine	Troponin T reference < 0.02 ng/ml	Imaging	Ischemic work-up
A	25	Male	Crohn's disease (no medications)	Chest pain 4-day post-2^nd^ vaccine	BNT162b2 Pfizer/BioNTech	Troponin T = 0.74 ng/ml	EKG: no ischemic changes Echo: left ventricular ejection fraction (LVEF) = 50% Cardiac MRI (cMRI): LVEF = 56%, consistent with myocardial edema (T2 mapping) and myocardial injury (focal late gadolinium enhancement (LGE)) with multiple foci in the midwall	Not applicable
B	28	Male	Obesity	Chest pain 3-day post-2^nd^ vaccine	BNT162b2 Pfizer/BioNTech	Troponin T = 1.42 ng/ml	EKG: lateral ST elevation myocardial infarction cMRI: LVEF = 56%, consistent with myocardial edema (T2 mapping) and myocardial injury (focal LGE) in the basal inferior, mid- to distal lateral/inferolateral walls	Coronary angiogram: no luminal irregularities
C	36	Male	Gastroesophageal reflux disease	Chest pain 2-day post-2^nd^ vaccine	mRNA-1273 Moderna/NIH	Troponin T = 0.35 ng/ml	EKG: lateral ST elevations Echo: LVEF = 55-60% cMRI: LVEF = 56%, consistent with myocardial edema (T2 mapping) and myocardial injury (focal LGE) in the mid- to distal inferolateral and lateral walls	Coronary angiogram: no luminal irregularities
